# Combination of Digital and Conventional Workflows in the CAD/CAM-Fabrication of an Implant-Supported Overdenture

**DOI:** 10.3390/ma13173688

**Published:** 2020-08-20

**Authors:** Michael Benno Schmidt, Angelika Rauch, Marcus Schwarzer, Bernd Lethaus, Sebastian Hahnel

**Affiliations:** 1 Clinic for Prosthodontics and Dental Materials Science, University of Leipzig, 04103 Leipzig, Germany; angelika.rauch@medizin.uni-leipzig.de (A.R.); sebastian.hahnel@medizin.uni-leipzig.de (S.H.); 2 Flemming Dental Tec GmbH, Dental Technology Center, 04317 Leipzig, Germany; marcus.schwarzer@flemming-tec.de; 3 Clinic for Oral and Maxillofacial Surgery, University of Leipzig, 04103 Leipzig, Germany; bernd.lethaus@medizin.uni-leipzig.de

**Keywords:** computer-aided design, dental implants, denture, complete, locator, overdenture, retention, prosthesis, work flow

## Abstract

Completely digital workflows for the fabrication of implant-supported removable restorations are not yet common in clinical dental practice. The aim of the current case report is to illustrate a reliable and comfortable workflow that reasonably merges conventional and digital workflows for the CAD/CAM-fabrication of implant-supported overdentures. The 53-year old patient was supplied with a digitally processed complete denture in the upper jaw and, simultaneously, with an overdenture supported by four interforaminal implants in the lower jaw. The overdenture included a completely digitally processed and manufactured alloy framework that had been fabricated by selective laser sintering. The case report indicates that digital manufacturing processes for extensive and complex removable restorations are possible. However, as it is currently not yet possible to digitally obtain functional impressions, future developments and innovations might focus on that issue.

## 1. Introduction

Recent innovations in computer-aided design (CAD) and computer-aided manufacturing (CAM) coincide with relevant changes in the workflow for the fabrication of prosthetic restorations. For fixed restorations, a fully digital workflow can be applied in numerous clinical settings. However, for complex removable restorations, digital workflows are neither fully established nor commonly applied. Nevertheless, there have been promising developments promoting a fully digital workflow in the production of complete dentures in the recent years [1,2]. For instance, there are workflows that use face scanners to minimise the number of appointments required until insertion of the prostheses to three, but the outcome of these workflows is not yet evidence-based in terms of accuracy and aesthetics [1,3]. 

Regarding the fabrication of complete dentures, the biggest problem associated with a fully digital workflow is the impression, because the functional margins have to be captured in a digital photograph. Current digital cameras are only able to properly digitalise attached gingiva [4]. However, the fabrication of complete dentures with sufficient retention requires a suction effect that is produced by the outer valve, which is generated by a slight displacement of the movable gingiva by the outer borders of the prosthesis in its vestibular area. To date, digital means cannot adequately replace this effect that is usually produced with the conventional functional impression and transferred into the master cast model. Thus, regarding digital workflows in the production of complete dentures, the most innovative approaches can currently be found in the laboratory. Digital laboratory workflows have their origin either in the digitalization of the functional impressions or the mounted cast models. In the various laboratory workflows currently available, the shape and position of the denture teeth can be modified via CAD before the digitally constructed dentures are forwarded as a dataset to the CAM process. The latter includes both subtractive (milling of the denture base and teeth from round blanks in milling machines) and additive (3D-printing of the denture base and teeth in a 3D-printer) approaches. Complete dentures fabricated in subtractive approaches feature an improved fit, since the conventional production process involves polymerization shrinkage of the acrylic resin. Digital laboratory processes omit polymerisation shrinkage as the dentures are milled from pre-polymerised blanks, which results in improved suction and retention of the denture [5,6]. These advantages also coincide with improved wearing comfort and reduce the incidence of ulcers [5]. It has also been highlighted that the pre-polymerised resin materials feature an enhanced fracture resistance [7]. Moreover, the surfaces of CAD/CAM-manufactured materials are smoother than those of conventionally processed materials and feature an increased surface hydrophilicity, which could result in less microbial adhesion [8]. Interestingly, no significant difference in the release of residual monomers has been identified between CAD/CAM-processed and conventional heat-cured denture base resins; however, it might be possible that other conventional denture base resins, such as cold-curing materials, feature a higher release of residual monomers [9]. The availability of the digital dataset is another advantage of the digitally processed denture, as it can be easily stored and refabricated in case of loss or fracture. This circumstance might be beneficial, for instance, in geriatric settings and patients with reduced adaptability towards new restorations. 

It is clear that the individual prosthetic treatment concept has to be selected in dependence on the individual requirements in relation to the prognostic factors of the various treatment options [10]. In edentulous patients, numerous prosthetic treatment options exist, including complete dentures, implant-supported fixed prostheses, and implant-supported removable prostheses with different attachment systems (stud, double crown, bar, or ball). Regardless of the attachment system, survival rates of implants and prosthetic restorations are high, yet in splinted prosthetic constructions, fewer complications were observed than, for instance, in constructions including stud attachments [11,12]. In the edentulous lower jaw, the most common problems associated with complete dentures include insufficient stability and retention. In these cases, implant-supported overdentures are commonly accepted as a standard treatment option [13,14,15] with high survival rates [16,17,18]. Implant-supported overdentures are designed and fabricated in accordance with the principles applied for complete dentures. However, digital workflows are not yet established for the fabrication of overdentures, and only limited information is available regarding the combination of conventional and digital workflows for the fabrication of these restorations. With regard to these aspects, the current case report illustrates a joined conventional and digital workflow for the fabrication of a complete denture in the upper jaw and overdenture supported by four implants in the lower jaw. 

## 2. Case Report 

A 53-year-old edentulous male patient introduced himself for prosthetic treatment at the Clinic for Prosthodontics and Dental Materials Science at the University of Leipzig. The patient’s general health condition was poor as a result of several diseases including liver transplantation and chronic kidney failure. The removable denture prostheses in the upper and lower jaw were severely worn and featured only insufficient retention. The patient demanded a significant improvement in denture retention, particularly in the lower jaw, and was informed about the various treatment options including complete dentures, implant-supported overdentures, or fixed implant-supported prostheses. The patient decided on an implant-supported overdenture connected with four Locator abutments in the lower jaw and a conventional denture prosthesis in the upper jaw. 

The insertion of the implants was planned with a wax-up that was finally transferred into an x-ray template. Digital volume tomography was employed by using the template to plan the positions and types of four interforaminal implants (Bone Level, 3.3 mm × 10.0 mm, Straumann, Basel, Switzerland). Subsequent to the approval of the implant positions, a surgical drilling guide was fabricated from the data. Correct planning of implants is highly important because malpositioned implants can cause esthetical, biological, as well as technical failures [19]. 

**Figure 1:**
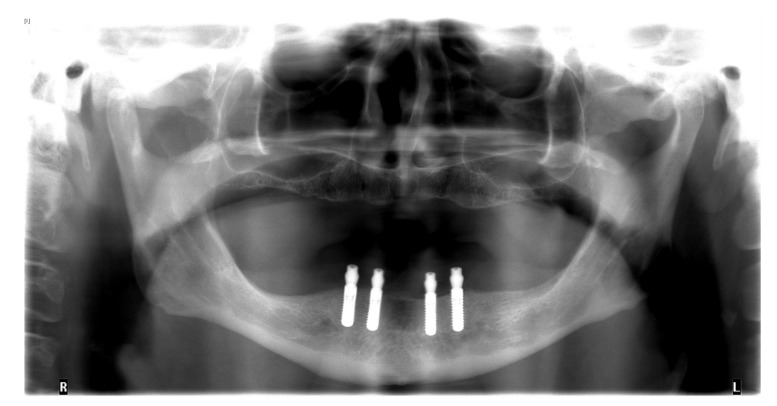
Orthopantomogram after insertion of four implants in the lower jaw.

**Figure 2:**
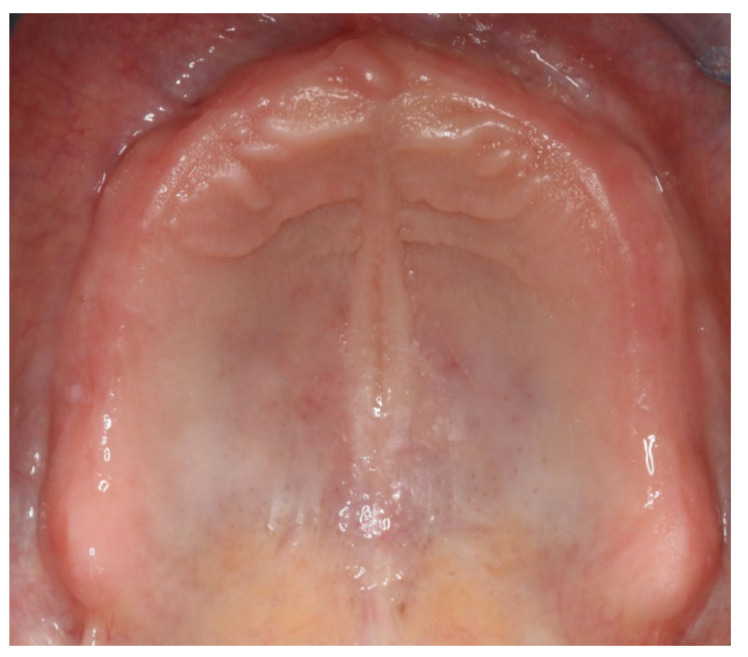
Intraoral photograph of the edentulous upper jaw.

**Figure 3:**
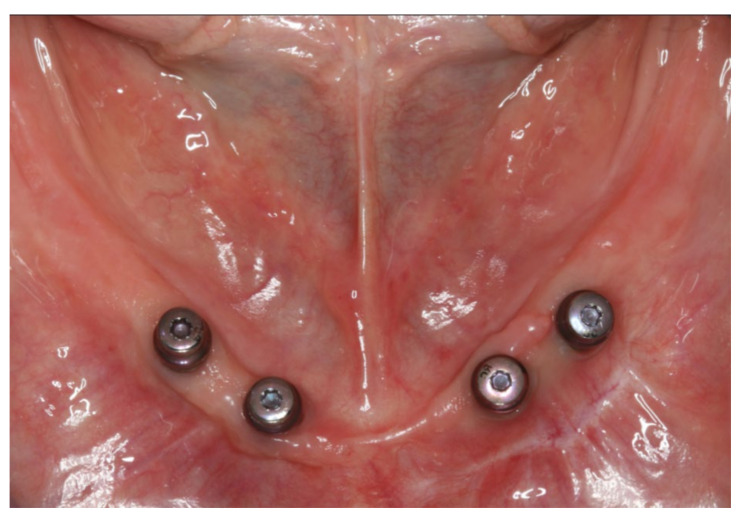
Intraoral photograph of the lower jaw with four interforaminal implants supplied with healing caps.

**Figure 4:**
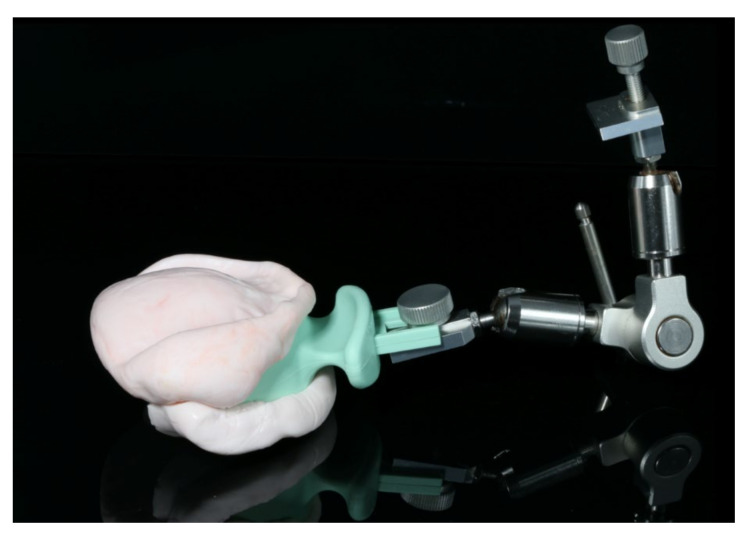
Preliminary bite registration taken during the first appointment.

**Figure 5:**
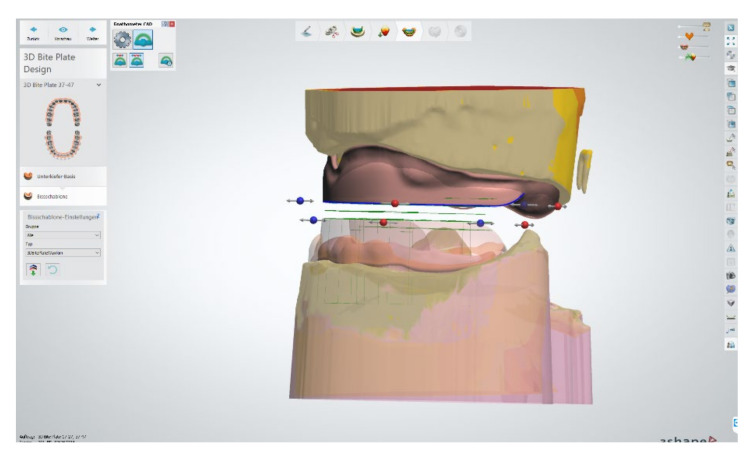
Digitally designed trays on the digitalised cast models.

**Figure 6:**
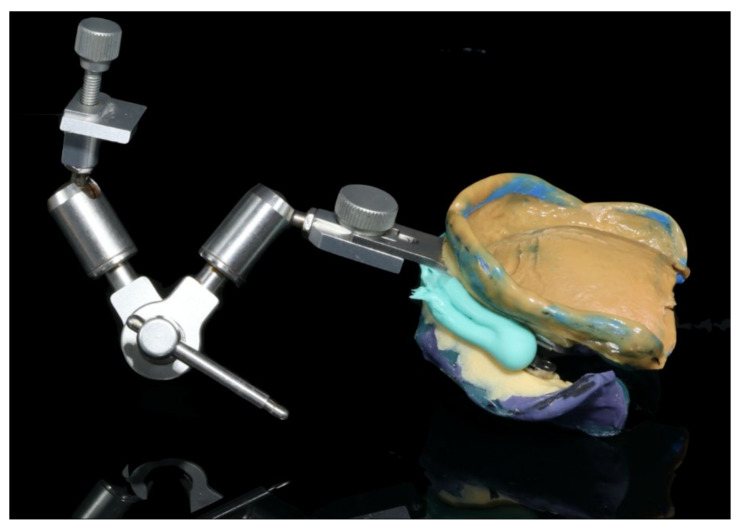
Functional impressions of the upper and lower jaw in combination with centric registration and face bow record.

**Figure 7:**
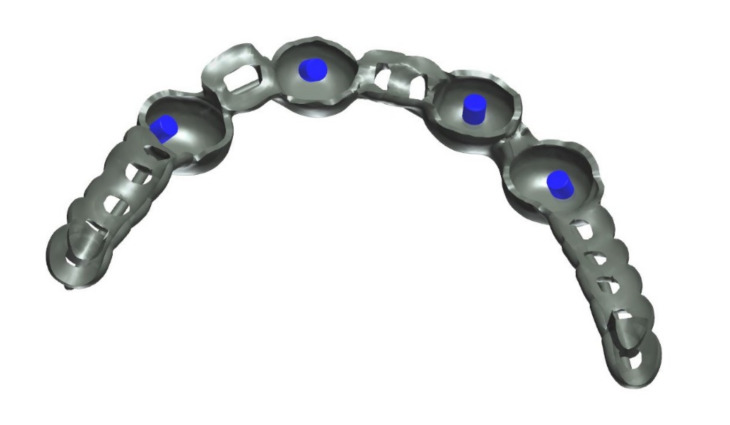
Close-up screenshot of the digitally constructed alloy framework.

**Figure 8:**
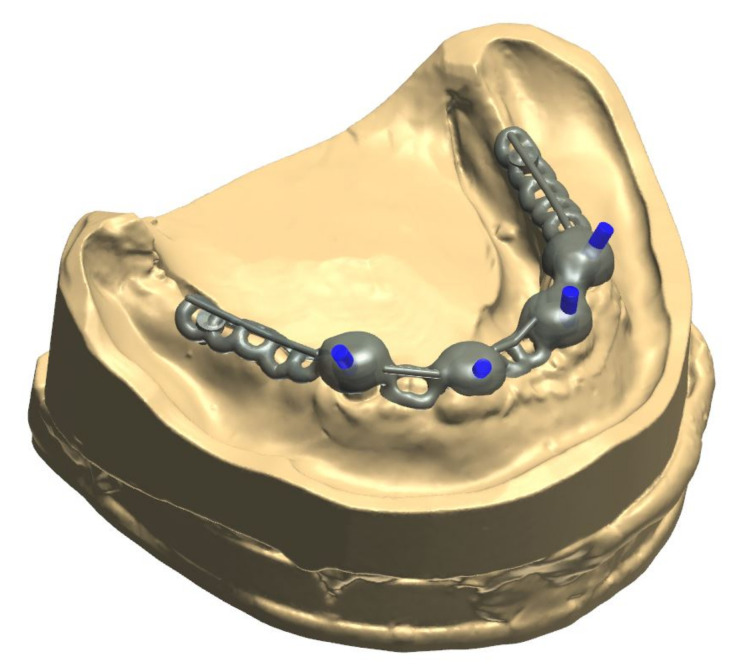
Digitally constructed alloy framework on the digital model of the lower jaw.

**Figure 9:**
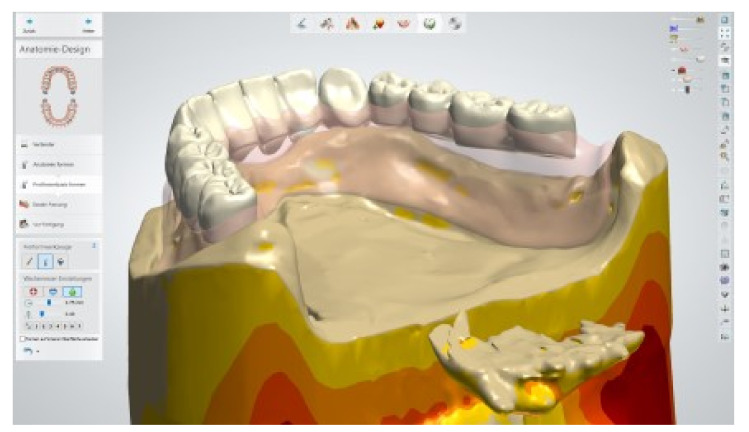
Positioning of denture teeth on the digitally blocked-out alloy framework in the lower jaw.

**Figure 10:**
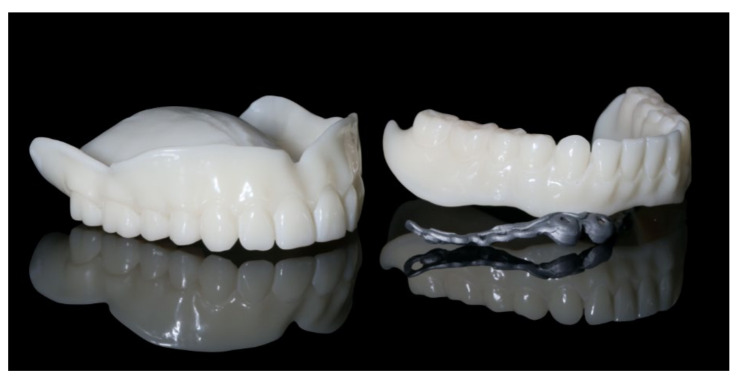
Additively manufactured try-in dentures for the upper and lower jaw and selectively laser sintered (SLS) alloy framework.

**Figure 11:**
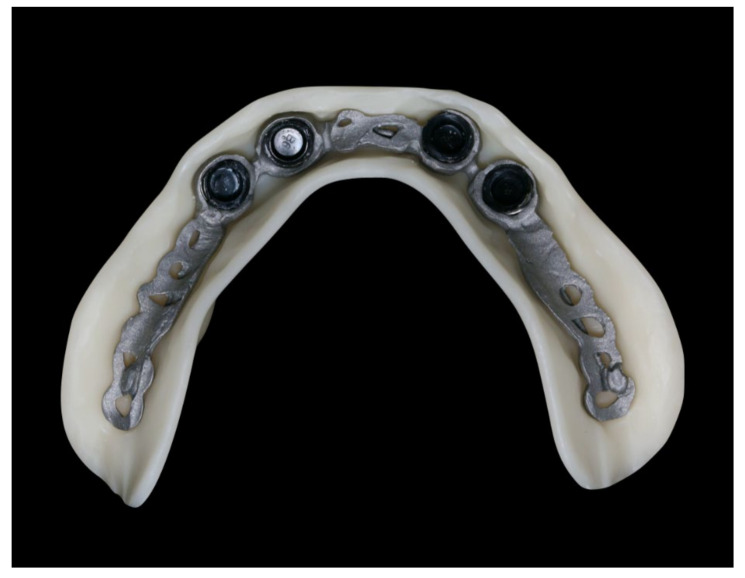
The digitally processed and fabricated alloy framework perfectly fits into the 3D-printed try-in denture in the lower jaw. As no further adjustments were necessary, the digital process simplified both the clinical and laboratory process.

**Figure 12:**
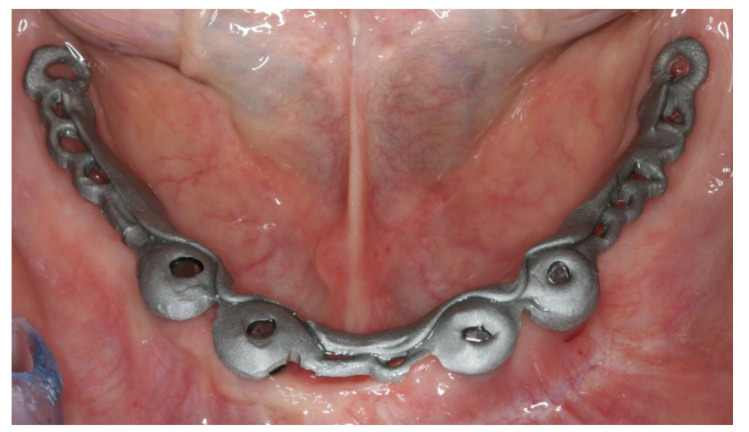
Alloy framework seated on the stud attachments in the lower jaw during try-in.

**Figure 13:**
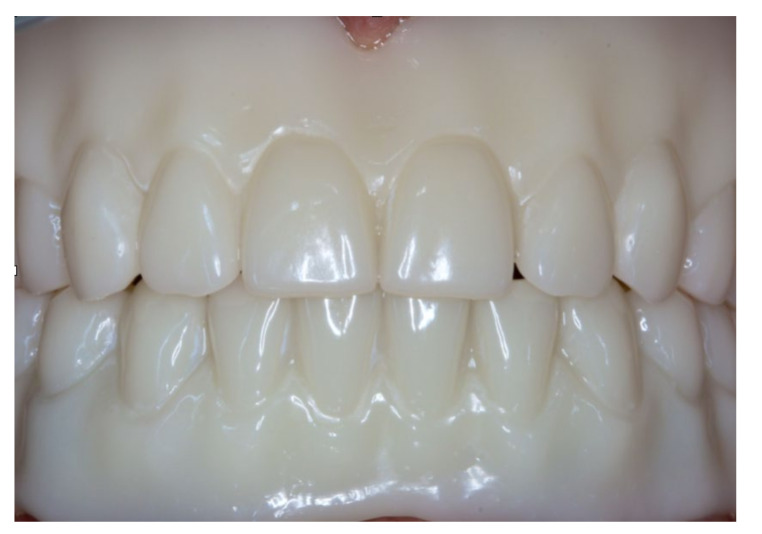
Anterior view of the try-in dentures in the upper and lower jaw during try-in.

**Figure 14:**
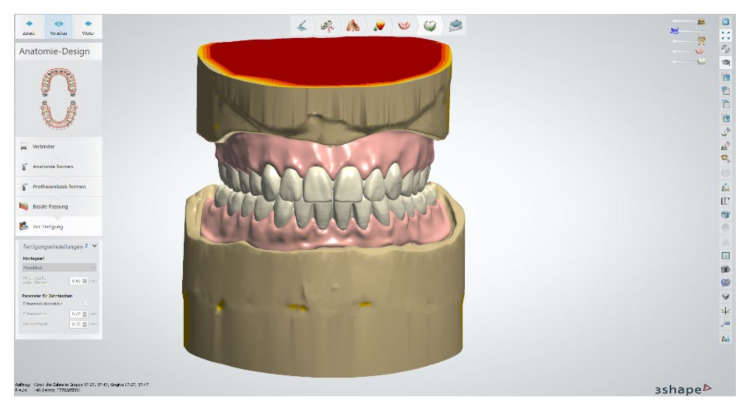
Construction of the definitive completed dentures in the upper and lower jaw.

**Figure 15:**
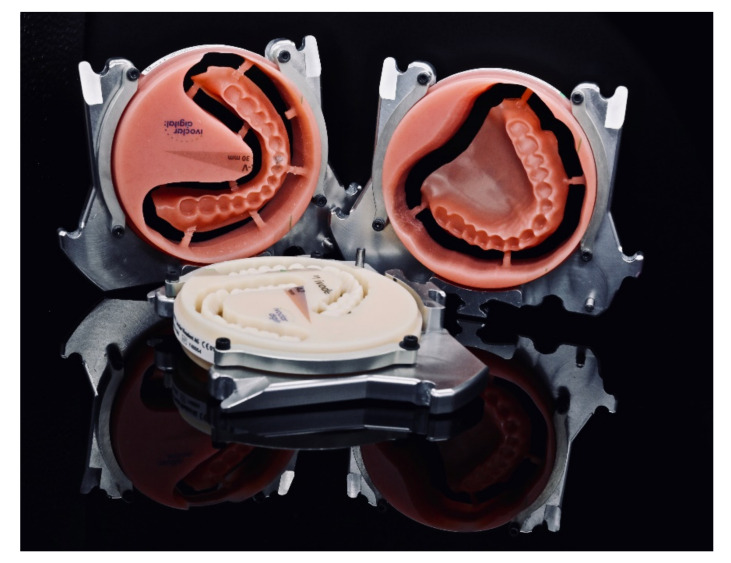
Denture bases and denture teeth milled from pre-fabricated blanks.

**Figure 16:**
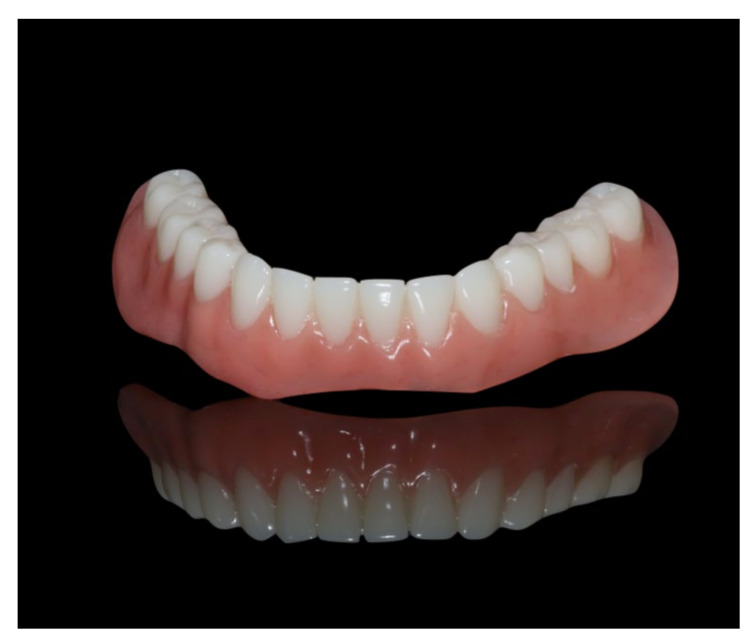
Polished implant-supported overdenture in the lower jaw ready for insertion.

**Figure 17:**
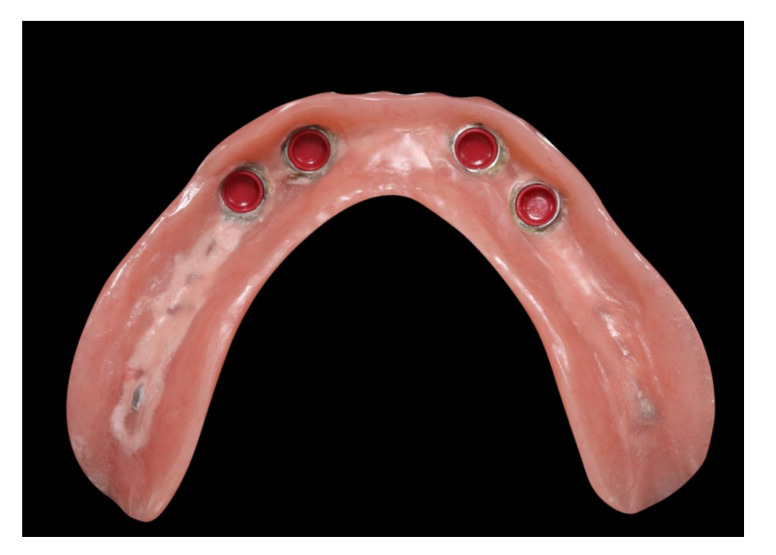
Basal view of the implant-supported overdenture in the lower jaw and its supporting framework equipped with intermediate retention inserts.

**Figure 18:**
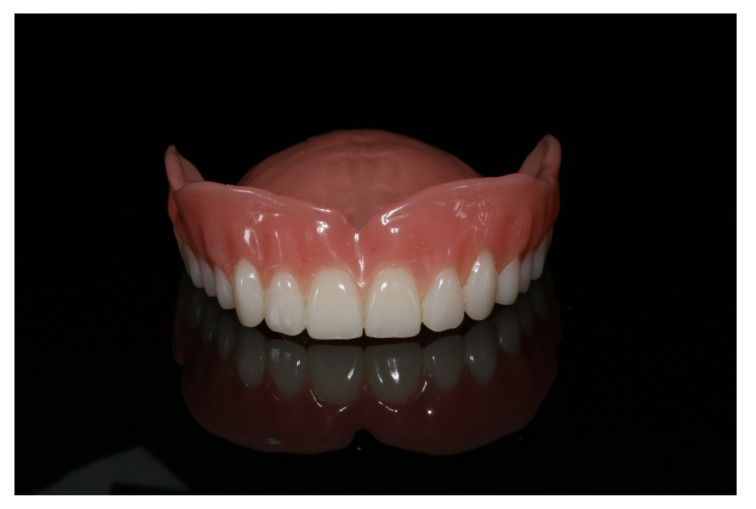
Polished complete denture in the upper jaw ready for insertion.

The healing time for the dental implants was four months. Subsequent to second-stage surgery and insertion of healing abutments (Figure 1), vestibular plastic surgery was performed employing an implant-supported dressing plate to produce sound and robust gingival tissues (Figure 2 and Figure 3). The first impressions were taken by using silicone-based impression materials (Kneton, Erkodent, Pfalzgrafenweiler, Germany) for the upper jaw and alginate for the lower jaw. The silicone-based material features constant mechanical properties and an extended setting time which is favourable for border moulding. For the lower jaw, an alginate impression material was used because it is easy to remove from the impression posts, which were inserted to indicate the implant position. A preliminary bite registration was performed using a special technique (Centric Tray, Ivoclar Vivadent, Schaan, Liechtenstein) (Figure 4) that simultaneously determines the preliminary vertical dimension. Cast gypsum models were prepared and digitalised in the dental laboratory and individual trays were designed and printed (3Shape, Copenhagen, Denmark; Flemingprint tray by DETAX, Ettlingen, Germany; Asiga MAX UV 3D Printer, Sydney, Australia) (Figure 5). An intraoral registration device based on needle-point tracing (Gnathometer M, Ivoclar Vivadent) was mounted on the trays. Following parallelisation of the plane of the upper jaw to the Camper`s plane and the bipupilar line, the vertical dimension was phonetically verified. The trays were used to take functional silicone impressions of the upper jaw in accordance with the suction effective mandibular complete denture (SEMCD) technique described by Abe [20,21]. Following border moulding, a functional impression of the lower jaw was taken by using polyether (Impregum, 3M, Saint Paul, MN, USA) impression material to transfer the implant position into the dental laboratory. Prior to this, the centric relation was determined with the intraoral registration device fixed onto the trays (Figure 6). In the dental laboratory, impressions and centric registration were digitalised. Subsequent to preparation of an implant master gypsum model with inserted stud attachments (Locator, Straumann), the alloy framework supporting the overdenture and housing of the attachment patrices was designed with CAM and produced using selective laser sintering (SLS) (FD MOG Flussfisch RPD, Flussfisch, Hamburg, Germany) (Figure 7 and Figure 8). Complete denture prostheses were designed in the upper and lower jaw by using CAD/CAM software (3Shape Dental system 2019 premium, Full denture design modul, Kopenhagen, Denmark) and the concept of a bilaterally balanced occlusion. The dataset was used to print try-in templates (Flemmingprint temp made by DETAX, Ettlingen Germany; Asiga MAX UV 3D Printer, Alexandria, Australia) (Figure 9). During the next appointment, the framework (Figure 10, Figure 11 and Figure 12) and templates were tried in separately (Figure 13) and the patient was satisfied with function and aesthetic appearance. Afterwards, the framework was stained with a pink opaquer, and the removable dentures and teeth were milled from pre-polymerised blanks (IvoBase CAD and SR Vivodent CAD, Ivoclar Vivadent, Schaan, Liechtenstein) using the dataset provided by the software (Figure 14). Subsequent to the milling process (vhf S2, vhf camfacture AG, Ammerbuch, Germany) (Figure 15), the teeth were inserted by hand. The polymeric overdenture and its supporting alloy framework were joined with a polymer (Pala X-Press, Kulzer, Hanau, Germany) and polished (Figure 16, Figure 17 and Figure 18).

**Figure 19:**
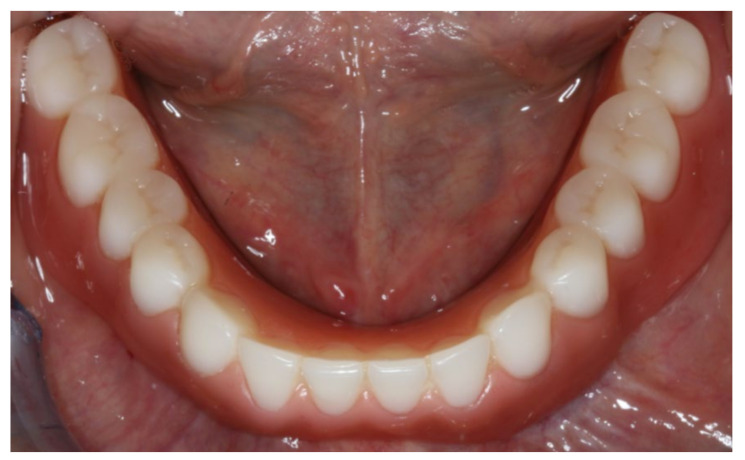
Inserted implant-supported overdenture in the lower jaw.

**Figure 20:**
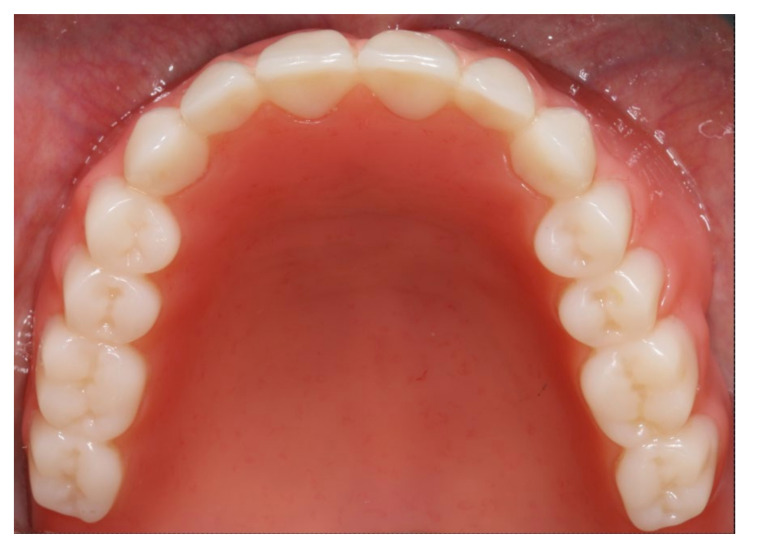
Inserted complete denture in the upper jaw.

**Figure 21:**
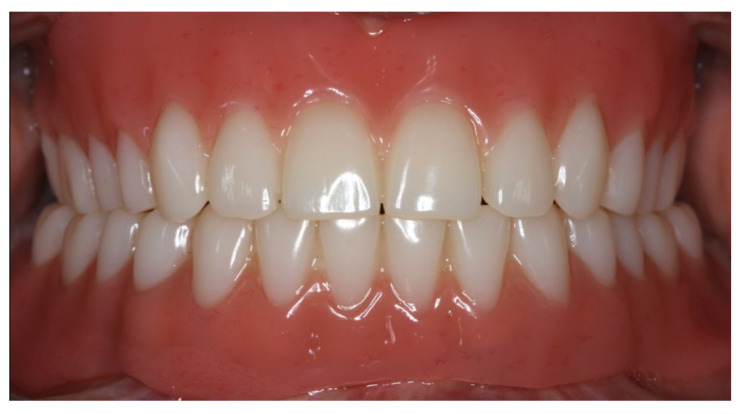
Anterior view of the inserted dentures in centric occlusion.

During the final appointment, the Locator abutments were tightened with torque (0.35 Nm) and the dentures were inserted. The attachment patrices were equipped with inserts producing intermediate retention (green). Occlusion, extension, retention, and aesthetics were carefully controlled and adjusted where appropriate (Figure 19, Figure 20 and Figure 21). Both dentures featured excellent retention and fit. Finally, the patient was instructed regarding insertion, removal, and maintenance of the dentures. After one week, the patient was satisfied with the new prostheses and no complications were reported or identified.

## 3. Discussion

**Figure 22:**
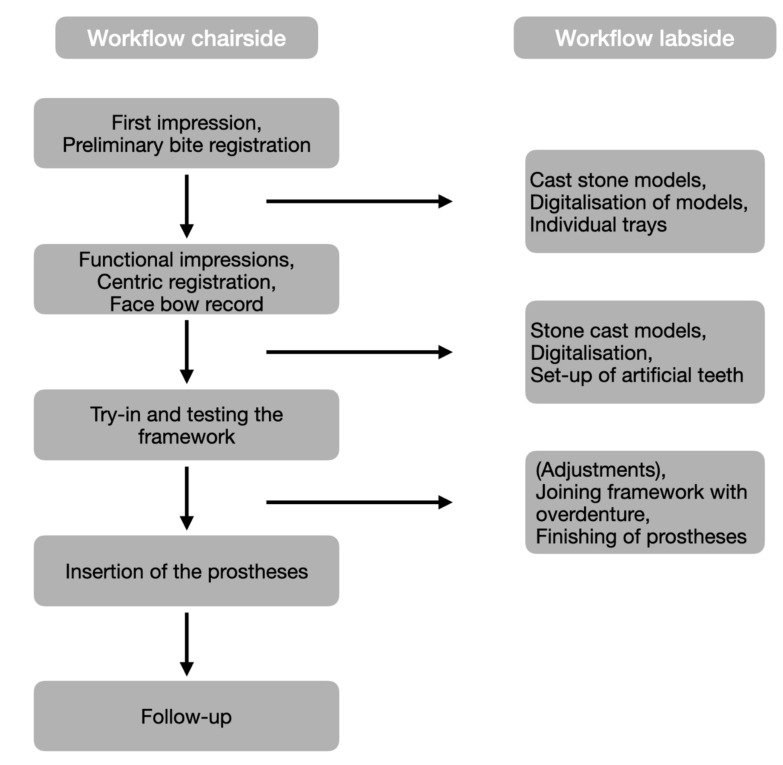
Flow-chart of the combined workflow.

The current case report illustrates a modern laboratory approach for the combination of conventional and digital workflows during the fabrication process of implant-supported overdentures (Figure 22). Regarding implantology, the insertion of four interforaminal implants is a standard treatment option for the edentulous lower jaw. Other commonly accepted treatment options include the insertion of two implants [9,10], yet a recent review has highlighted that the survival rate of four implants in the edentulous lower jaw is higher than for fewer implants [22]. In the current patient, the upper jaw was supplied with a gum-supported complete denture, which produces less chewing forces than implant-supported fixed or removable restorations. Thus, the authors decided to supply the implants in the lower jaw with stud attachments rather than bar or double crown attachments, or a fixed restoration. This procedure also reduced laboratory costs and provided the option to easily adjust the retention of the overdenture by changing the inserts, though the authors are aware that overdentures retained by stud attachments require frequent maintenance [23,24].

Intraoral scanners are successfully used for various purposes in the field of dentistry, including the fabrication of fixed dental prostheses on teeth and implants [25,26]. While digital technologies are regularly necessary to process novel materials that cannot be processed with traditional techniques (e.g., zirconia) [27], studies have underlined that treatment times can be significantly reduced and patients prefer intraoral scanning in comparison to conventional impression procedures [26,28]. Currently, oral scanners are not able to digitalize all relevant parts of the edentulous jaw [4], since functional movements cannot be sufficiently recorded. Thus, the functional impression remains to be completed in an analogue manner by the dentist. The authors followed the SEMCD protocol for the fabrication of the complete denture, which requires four treatment appointments, including first impressions and preliminary bite registration (I), functional impressions and intraoral registration (II), try-in (III), and insertion (IV). In the current study, the protocol was further amended by including the try-in and insertion of a CAD/CAM-fabricated alloy framework without increasing the number of required appointments. In the near future, the application of face scanners could further reduce the number of required appointments for the fabrication of complete dentures to three [2]. However, the accuracy that can be achieved by combining and matching different data sets gathered by 3D face scanners is still limited [29]. Future developments in optical scanning devices might also allow the differentiation between fixed and movable gingiva, which might pave the way for a fully digital process to fabricate complete dentures and might even further reduce the number of appointments required. However, it has also been reported that reducing the number of appointments in the process of manufacturing a complete denture might lead to mistakes (e.g., in the determination of the vertical dimension of occlusion) and might coincide with patient dissatisfaction [30,31]. These studies revealed that bite registration is a crucial step in the fabrication process of a complete denture. To improve patient satisfaction, the authors decided to perform impression and bite registration in an analogue approach. However, it has been reported that workflows combining both conventional border moulding and digital scanning of the gingiva coincided with no relevant increases in unscheduled follow-up appointments [32]. Based on their own experiences, the authors recommend the inclusion of a try-in step in the fabrication process. Try-in templates manufactured by 3D-printing may be used as travel prostheses. In order to reduce treatment costs in the current case, the try-in templates were printed from a single white resin. More complex regimes may also employ different resins with different colours to produce templates differentiating the colour differences between denture base and teeth. 

The alloy framework employed in the current case was manufactured by selective laser sintering. Recent studies have highlighted that frameworks for removable dentures that had been fabricated by SLS feature an adequate precision [33,34,35,36]. However, to estimate the long-term outcome of these constructions, more clinical studies are required [22].

The authors are aware that digital dentistry is rapidly evolving. Due to the tremendous turnover rate, it is, however, often difficult to introduce these innovations into the daily clinical routine. Against this background, the current case report illustrates a reliable and comfortable workflow that can be followed by prosthodontists to almost completely digitally produce complete dentures and implant-supported overdentures. It is likely that future innovations such as advances in 3D printing and face scanning will further digitalise the workflow.
